# Structure and Dynamics of Dinucleosomes Assessed by Atomic Force Microscopy

**DOI:** 10.1155/2012/650840

**Published:** 2011-10-23

**Authors:** Nina A. Filenko, Dmytro B. Palets, Yuri L. Lyubchenko

**Affiliations:** ^1^Department of Pharmaceutical Sciences, University of Nebraska Medical Center, Omaha, NE 68198-6025, USA; ^2^Department of High Technologies, The International Solomon University, 1b Sholudenko Street, Kyiv 01135, Ukraine; ^3^Department of Application Services, Infopulse Ukraine LLC, 24 Polyova Street, Kyiv 03056, Ukraine

## Abstract

Dynamics of nucleosomes and their interactions are important for understanding the mechanism of chromatin assembly. Internucleosomal interaction is required for the formation of higher-order chromatin structures. Although H1 histone is critically involved in the process of chromatin assembly, direct internucleosomal interactions contribute to this process as well. To characterize the interactions of nucleosomes within the nucleosome array, we designed a dinucleosome and performed direct AFM imaging. The analysis of the AFM data showed dinucleosomes are very dynamic systems, enabling the nucleosomes to move in a broad range along the DNA template. Di-nucleosomes in close proximity were observed, but their population was low. The use of the zwitterionic detergent, CHAPS, increased the dynamic range of the di-nucleosome, facilitating the formation of tight di-nucleosomes. The role of CHAPS and similar natural products in chromatin structure and dynamics is also discussed.

## 1. Introduction

The formation of nucleosomes is the first stage of DNA packing into chromatin, followed by the assembly of the “beads-on-a-string” nucleosomal array into compact chromatin fibers (e.g., [1] and references therein). H1 histone is a key player in the formation of the 30-nm-thick fibers (e.g., [2]); however, interactions between the nucleosomal particles contribute to the assembly process as well. 

Although the molecular mechanisms behind the formation of higher-order chromatin structures remain unclear, work employing model systems has shown that *in vitro* reconstituted nucleosome arrays containing only DNA and core histone proteins undergo the same initial salt-dependent condensations as native chromatin [1, 3]. In solutions containing physiological concentrations of mono- and divalent cations, nucleosome arrays spontaneously fold into structures with the same hydrodynamic shape as the 30-nm-diameter chromatin fiber [1, 4]. This implies that the primary protein determinants defining these structures reside within the core histone proteins. Indeed, in the early crystallography work of Luger et al. [5], it was shown that the K16 to N25 segment of H4 makes extensive contacts with an H2A-H2B dimer of an adjacent particle. This H4 region, K16 to N25, makes multiple hydrogen bonds and salt bridges between its basic side chains (K16, R19, K20, R23) and acidic side chains of H2A (E56, E61, E64, D90, E91, E92) and H2B (E110). These structure-based predictions were supported with studies [6–8] of recombinant core mutants. They showed that interaction between the H4 N-terminal domain (NTD) and a surface-exposed H2A region on a neighboring nucleosome is required for assembly of folded 30-nm secondary structures. More importantly, nucleosomal arrays lacking all core histone NTDs are unable to fold into the 30-nm secondary chromatin structures [3, 9], even in the presence of bound linker histones [10]. This data shows that interaction between nucleosomes in the absence of H1 takes place. Results indicate that all core histone N-terminal domains (NTD) participate in the oligomerization process and that the NTDs function additively and independently [11]. According to the Monte Carlo simulations [12], the H4 tails mediate the majority of the internucleosomal interactions, followed by the H3, H2A, and H2B tails in decreasing order. The H4 histone role was also studied separately and was found that the N-terminal end of the H4 tail participates in intra- and internucleosomal interactions with protein and DNA during folding and oligomerization of nucleosome arrays [13]. 

Atomic force microscopy (AFM) was applied in [14] to study interactions between nucleosomes within the assembled “beads-on-a-string” nucleosomal array in the absence of H1 histone. The experimental data were supplemented with computer simulations. The results led to the conclusion that attractive interactions between the nucleosomes existed which led to array compaction. In recent work [15], the interaction between nucleosomes was studied with the use of FRET. With this technique, transiently formed dinucleosomes with the lifetime ~1 s were detected. The acetylation of histones and the presence of divalent cations increased the stability of dinucleosomes. 

Here, we apply AFM imaging to directly characterize the formation of tight dinucleosome complexes with the dinucleosome design. We showed that such complexes are formed but with low efficiency. The data are in line with the dynamic nature of dinucleosomes, in which the distance between the nucleosomes varies in a broad range, enabling the formation of close nucleosome-nucleosome contacts. We demonstrate the importance of environmental conditions to these dynamics and characterize the role of the ionic detergent CHAPS.

## 2. Materials and Methods

### 2.1. Preparation of Nucleosomal DNA

DNA for nucleosome assembly was generated by PCR using the plasmid pGEM3Z-601 as a template, which codes for a high-affinity nucleosome positioning sequence [16]. The PCR reaction (33 cycles of 94°C/30 s, 54°C/30 s, 72°C/30 s) was run in buffer containing 2.5 mM MgCl_2_, 0.15 mM dNTPs, and 0.016 U/*μ*L of Taq DNA polymerase. Two PCR reactions were run with forward and reverse primers, respectively, set 1, primers CGGCCAGTGAATTGTAATACG and CGGTACGCTGGG TATGTGATGGACCCTATACG, and set 2, primers GCCATCCCCAGCCGGCAAGGTCGCTGTTCAAT and CGGGATCCTAATGACCAAGG. These reactions created products, which incorporated nonpalindromic BseYI restriction site at the ends. After restriction digest with BseYI and ligation with T4 DNA ligase, a DNA template of 560 bp was obtained (see Figure S1 in Supplementary Material available online at doi:10.1155/2012/650840), which contained two nucleosome positioning sequences with a 60 bp linker between them and arms of 127 and 79 bp in length.

### 2.2. Histone Octamer Assembly and Purification

Histone octamers were assembled as follows [17]. Octamers were separated from tetramer and dimer fractions with size-exclusion chromatography (SEC) with Superdex 200 PC 3.2/30 column (GE Healthcare) at 4°C. SEC fractions were analyzed for purity and histone stoichiometry using SDS-PAGE. The gel was stained using Coomassie Blue stain. Fractions containing histones H2A, H2B, H3, and H4 in approximately equal ratios were pooled and concentrated by centrifugation at 10,000 g.

### 2.3. Nucleosome Refolding

Dinucleosome particle samples (di-NCP) were prepared as described earlier [18, 19]. Briefly, histone octamers and DNA containing the nucleosome positioning sequence were mixed in equimolar concentrations in 2 M NaCl and kept for 30 min at RT. A dilution series was prepared using 10 mM Tris HCl to produce final concentrations of 1 M, 0.67 M, and 0.5 M NaCl. Diluted samples were kept at 4°C for 1 h before dialysis against one change of volume of 0.2 M NaCl overnight. Nucleosomes were concentrated using Microcon centrifugal filter devices, MWCO 10,000 at 7,000 g for 10 min at 4°C and dialyzed against one change of 200 mL of buffer containing 10 mM Hepes-NaCl, pH 7.5, and 1 mM EDTA for 3 h at 4°C.

### 2.4. Atomic Force Microscopy

Freshly cleaved mica was modified with a 167 *μ*M solution of 1-(3-aminopropyl)-silatrane (APS) for 30 min at room T to make APS-mica as described previously in [19–21]. The nucleosome stock solution was diluted into 10 mM Tris-HCl, pH 7.5, 4 mM, MgCl_2_ buffer, and 5 *μ*L of the solution was deposited on APS-treated mica for 3 minutes, washed with deionized water, and dried under argon flow. AFM images were collected on an MM AFM/NanoScope IIId system (Bruker-Nano/Veeco, Santa Barbara, Calif, USA) as described in [19, 20, 22].

### 2.5. Measurement of Nucleosome Parameters

**Figure 1:**
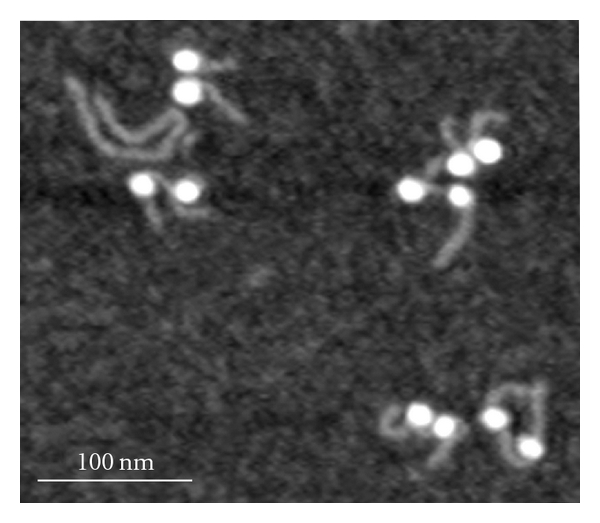
AFM images of dinucleosomes obtained with the use of APS mica. AFM images were acquired with the use of the MM AFM/NanoScope IV system operating in Tapping Mode.

The samples deposited on APS mica were analyzed with FemtoScan software. The following 5 initial parameters were measured: length of each DNA arm, angle between arms (interarm angle), height of the nucleosome core particle, and diameter as the width of the nucleosome core particle at half height. The length of DNA was measured with FemtoScan software using the “curve” parameter. The length of wrapped DNA was measured by subtracting the sum of both DNA arms and the linker from the length of free DNA measured on the same sample (see Figure 1). The length of the linker DNA was measured from the center of one nucleosome along the linker to the center of another nucleosome minus 5 nm (the size of the nucleosome particle itself).

## 3. Results

### 3.1. Visualization of Dinucleosomes with AFM

The di-NCP sample was imaged similarly to the mononucleosome sample described earlier [18–22]. Diluted samples (0.8 nM concentration in 10 mM Tris-HCl, Ph 7.5, and 4 mM MgCl_2_ buffer) were deposited on APS mica and after drying, imaged in air at ambient conditions. Figure 1 shows a typical AFM image of the dinucleosome sample. The nucleosomes appear as two bright globular features (blobs) separated with a DNA region (linker) with two free DNA arms at each nucleosome. The yield of dinucleosomes in the sample is high. The image in Figure 1 has 6 dinucleosomes and only one free DNA.

### 3.2. Nucleosome Wrapping in the Dinucleosomes

**Figure 2:**
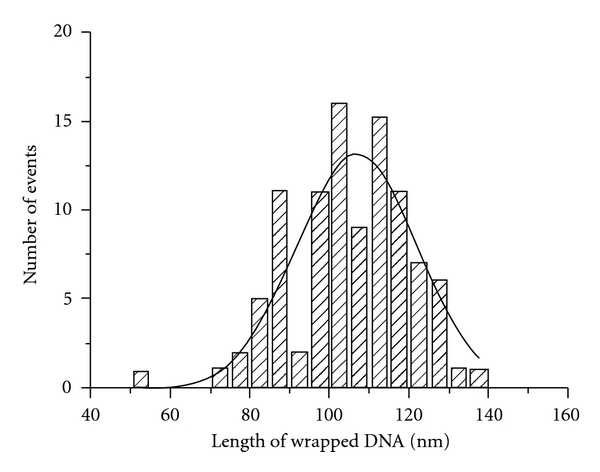
The length distribution for wrapped DNA for all dinucleosome molecules in the population. The value of total DNA wrapped around both octamers was calculated by subtracting the measured length of two arms and a linker from the total DNA length, as described in Section 2. The maximum of the Gaussian corresponds to 106.6 ± 2 nm.

Images as shown in Figure 1 were used for assessing the degree of nucleosome wrapping. This value was calculated from the measurements of the lengths of the DNA arms and the linker DNA as described in Section 2. The histogram for this value is shown in Figure 2. The distribution is rather broad with a maximum for the Gaussian = 107 nm. The expected length of wrapped DNA for the di-NCP is 147 (bp) × 0.32 (nm/bp) × 2 = 94.08 nm. Due to the tip convolution effect, NCPs on the AFM images appear ~10 nm larger [19]. This effect leads to a decrease of the length of the measured unwrapped DNA by ~10 nm; therefore, given the convolution effect, the measured mean value of wrapped DNA is close to the expected value. At the same time, the distribution of the lengths is broad, ranging between ~86 nm and 127 nm (mean values). This suggests that nucleosomes are dynamic, enabling the formation of particles with varied amounts of DNA wrapping, which is in line with our previous study of mononucleosomes [18, 19, 22].

### 3.3. The Internucleosome Distance and the Interaction between Nucleosome Cores

To address the question of whether nucleosome core particles interact with each other in the absence of H1 histone, we measured two parameters of dinucleosomes, the length of the linker, and the shortest internucleosome distance. The first parameter will answer the question of whether the interaction occurs via sliding of nucleosomes. The second parameter will reveal whether the nucleosomes interact in space without sliding. 

**Figure 3:**
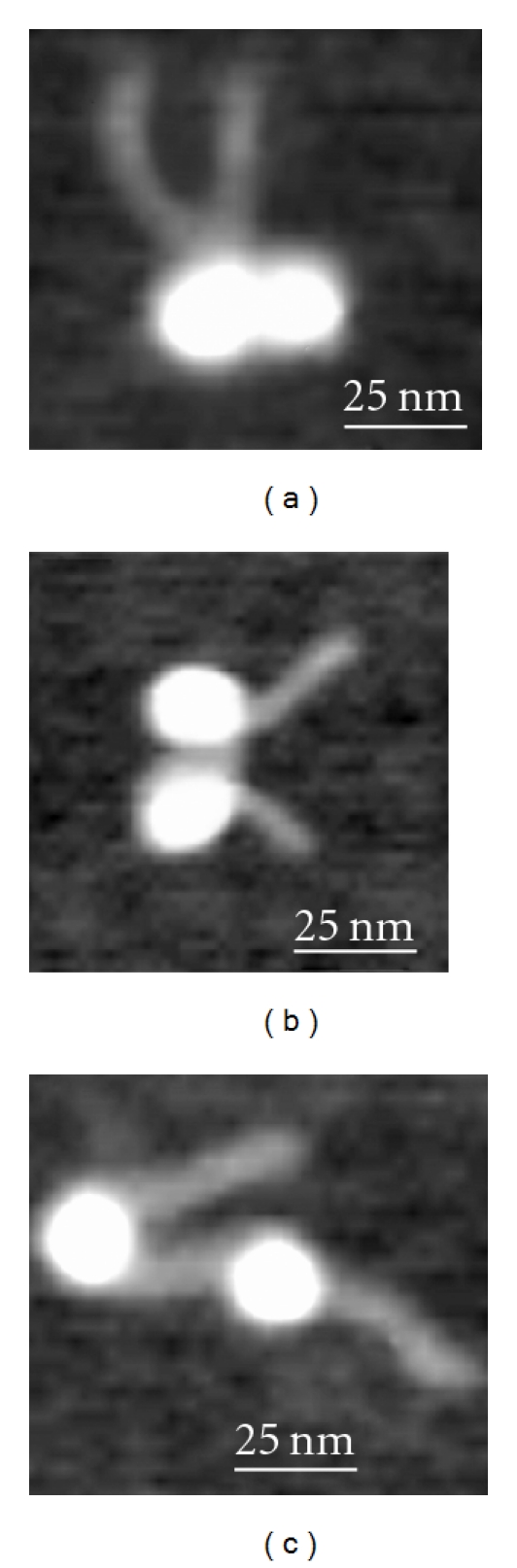
Selected images of dinucleosomes with different internucleosomal distances.

A subset of three images of dinucleosomes with different relative positions is shown in Figure 3. Frame “a” on this set corresponds to dinucleosomes with very small internucleosomal distances. The distance is considerably larger for the dinucleosome in Figure 3(b) (~17 nm) and the last image (Figure 3(c)) corresponds to a linker length ~37 nm. 

**Figure 4:**
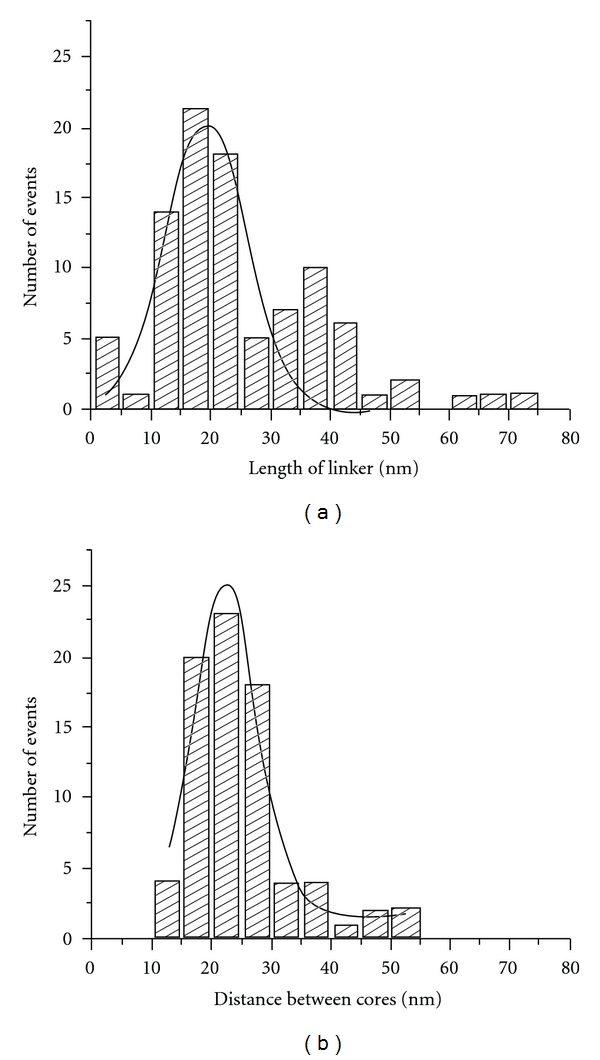
Distributions of the linker lengths (a) and the internucleosomal distances (b). Maximum of the Gaussian for the linker length is 18.9 nm. Maximum of the Gaussian for distances between cores is 22.2 nm.

The linker length measurements are shown in Figure 4(a). In addition to the main peak at the maximum ~19 nm, there is a minor peak at ~35 nm. The appearance of the second peak suggests that there is a population of partially unwrapped nucleosomes. This interpretation is in line with measurements of wrapped DNA. The most important finding of this analysis is that the measured linker length (~19 nm) is very close to the expected value, 19.84 nm. 

The shortest distance between the nucleosomes was obtained by measuring the center-to-center distance. The results are assembled in Figure 4(b). The dinucleosomes with visually low wrapping were excluded from this analysis. The histogram has a maximum at ~22 nm and a low percentage of nucleosomes with the internucleosomal distance of 10 nm. Thus, both measurements do not support the idea of interactions between the nucleosomes.

### 3.4. Effect of the Zwitterionic Detergent, CHAPS, on the Morphology of Dinucleosomes

Nucleosomes are unstable at low concentrations (nanomolar range), and detergents are used to stabilize them (e.g., [23]). We have shown recently that the zwitterionic detergent, CHAPS, considerably increases the stability of mononucleosomes at the nanomolar concentration and works in a broad range of ionic strengths [18]. Importantly, the sequence-specific positioning of the nucleosomes also changes in the presence of CHAPS. We analyzed the effect of CHAPS for the dinucleosome samples to understand whether CHAPS changes the stability and morphology of dinucleosomes. 

**Figure 5:**
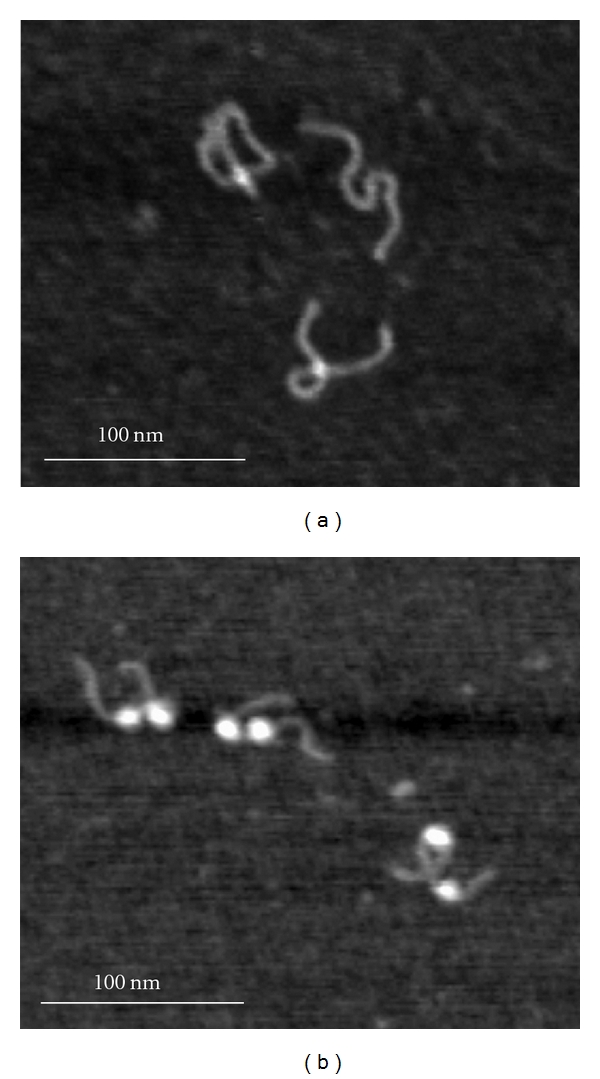
AFM images of the dinucleosome sample incubated in Tris-HCl buffer (10 mM Tris-HCl, pH 7.5, 4 mM MgCl_2_). The nucleosomes concentration was 0.8 nM, the sample was incubated at 4°C for 30 min without CHAPS (a) and with CHAPS (b).

Images in Figure 5 are the typical images obtained for the dinucleosome sample incubated in Tris-HCl buffer (10 mM Tris-HCl, pH 7.5, 4 mM MgCl_2_, pH 7.5) at the concentration 0.8 nM for 30 min in the absence of CHAPS (a) and in the presence of 1.6 mM CHAPS (b). The initial sample contained 85% of dinucleosomes and appeared under AFM as the sample shown in Figure 1. Incubation for 30 min (Figure 5(a)) leads to almost complete dissociation of the nucleosomes from the DNA template; therefore, only naked DNA is seen under AFM. In contrast, dinucleosomes remain intact if 1.6 mM CHAPS is present (Figure 5(b)). A similar analysis was performed for other incubation times, and the data are assembled in Figure S2. This graph shows that in the absence of CHAPS, nucleosomes dissociate rapidly with a characteristic time ~7.5 min, whereas in the presence of CHAPS, dinucleosomes are stable after incubation for 48 hours. Similar data were obtained for the mononucleosomes [18], suggesting that each nucleosome in the dinucleosome design behaves independently. 

We also measured the variation of the linker length over incubation time with 1.6 mM CHAPS. Initially at zero incubation time, both samples were indistinguishable with a linker length ~20 nm. Moreover, the distributions of the linker lengths for the control sample and the sample prepared in the presence of CHAPS were very close (Figure S3). The time-dependent measurements of the linker lengths for the CHAPS containing samples showed dramatic changes in the width distributions over time. The values change in the order of 11.4 nm, 22.3 nm, 23.1 nm, and 18.1 nm for times 0 min, 5 min, 2 hours, and 48 hours, respectively. The histograms for each incubation time are shown in Figure S4. These findings suggest that the sequence specific formation of nucleosomes, in the presence of CHAPS, is less stringent.

## 4. Discussion

The data obtained in this paper show that the direct interaction of two nucleosomes separated by a linker in the absence of H1 histone can occur, but the probability of such events is rather low. We were able to identify by AFM complexes with a small internucleosomal distance similar to the one shown in Figure 3(a). However, the yield of such complexes according to the statistical analysis is low (Figure 4(b)). At the same time, the AFM analysis shows that nucleosomes with a broad range of internucleosomal distances are formed, suggesting that nucleosomal particles in the di-nucleosomal construct are rather dynamic, enabling the formation of systems with different distances between the nucleosomes. The highly dynamic feature of nucleosomes was directly proven in our early work with mononucleosomes, in which the dynamics were observed directly with time-lapse AFM [19]. The findings in this paper are in line with our previous studies. Therefore, nucleosomes can translocate along the DNA, approach quite close to each other or move apart. The interaction between two nucleosomes was observed in [15], but these were free nucleosomes, and, in the complexes, the two nucleosomes were separated by approximately 2 nm. In addition, according to [15], the dinucleosome complexes are formed transiently, which is in line with our model of nucleosome dynamics. 

There are two models for the formation of tight dinucleosome complexes, translocation of nucleosomes, and their interaction in space via bending of the linker. The second mechanism requires substantial deformation of the linker. To make a close contact, a U-turn deformation is needed, which is problematic for a linker as short as 60 bp. Deformations of this kind require a segment with a random sequence 4 times larger in length [24]. Internucleosomal interactions were observed in [14], in which arrays of nucleosomes were used enabling the interaction of nucleosomes distantly located in the array. Therefore, we assume that translocation of the nucleosome is the mechanism by which close contacts between the nucleosomes are made. 

There are two models for nucleosome dynamics, sliding and site transfer requiring unwrapping of both arms of the nucleosome [25, 26]. Our previous findings [19] were in favor of the site transfer model. Indeed, the dynamics via unwrapping of both arms of the nucleosome was the only pathway imaged with time-lapse AFM directly. However, recently we applied high-speed AFM to directly image nucleosome dynamics [27]. These studies enabled us to observe transient sliding of the nucleosome at the subsecond time scale. The nucleosome moved from the original site-specific position and returned back to the same position. Note that this and our previous studies were performed with the use of the 601 sequence, characterized by a high affinity for nucleosome formation. Therefore, we assume that transient formation of tight di-nucleosomal complexes occurs via a sliding mechanism. 

According to the statistical analysis (Figure 4(a)), the lengths of the linker for different nucleosomes vary over 15 nm. However, the range is two times wider if CHAPS is added (Figure S4), suggesting that the dynamics of nucleosomes is much broader if CHAPS is present. We have shown recently (see [27]) that CHAPS facilitates nucleosome dynamics and sliding away from the 601 motif into regions with a random sequence occurs with the formation of stable nonspecific nucleosomal particles [18]. We hypothesized that in the presence of CHAPS, the stringency of the sequence specificity for nucleosome formation is lowered, enabling the formation of stably existing nucleosomes over the region away from the 601 motif [18]. The broad variability of the internucleosomal linker is in line with this hypothesis. 

Although CHAPS is not a natural product, it has similarity in structure with cholesterol. Cholesterol has the same conjugated 3 benzyl rings and 1 pentyl ring as CHAPS. Also, the content of cholesterol in nuclear membranes is about 10%. This suggests that the possible involvement of cholesterol, as a significant component of the nuclear membrane, in the regulation of chromatin dynamics should be taken into account. Importantly, a modified water-soluble cholesterol, cholesteryl sulfate, is a minor constituent of various cell types including erythrocytes [28] and spermatozoa [29]. It was shown that cholesteryl sulfate is also present in blood plasma [30]. We have shown [18] that cholesteryl sulfate also stabilizes nucleosomes and its participation in chromatin dynamics needs to be considered as well.

## Supplementary Material

The Supplementary Material section contains additional figures presenting the schematics of the DNA design, the data on kinetics of nucleosomes dissociation, and the results of the length measurements for the nucleosomes with and without CHAPS.
