# Supplementary material for: Structure and Dynamics of Dinucleosomes Assessed by Atomic Force Microscopy
Source: J Amino Acids. 2011 Oct 23;2012:650840. doi: 10.1155/2012/650840 (PMC3268019; doi:10.1155/2012/650840)
Supplement: Supplementary file 1 — The Supplementary Material section contains additional figures presenting the schematics of the DNA design, the data on kinetics of nucleosomes dissociation, and the results of the length measurements for the nucleosomes with and without CHAPS. [file 650840.f1.pdf]

SUPPLEMENTARY FIGURES

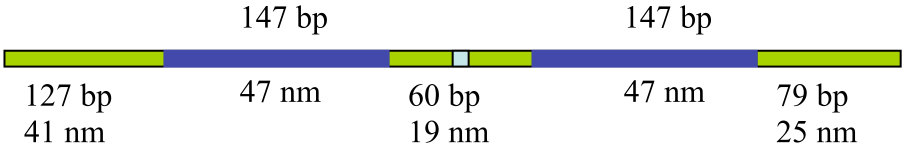

Figure S1. Schematics of the DNA design**.** The nucleosome-specific 601 sequences are shown in blue. The linker between these sequences and the terminal regions are shown in green. The lengths of each segments are shown in base pairs (bp) and nanometers (nm). *Bse*YI site at which two DNA fragments were ligated, is shown in light blue.

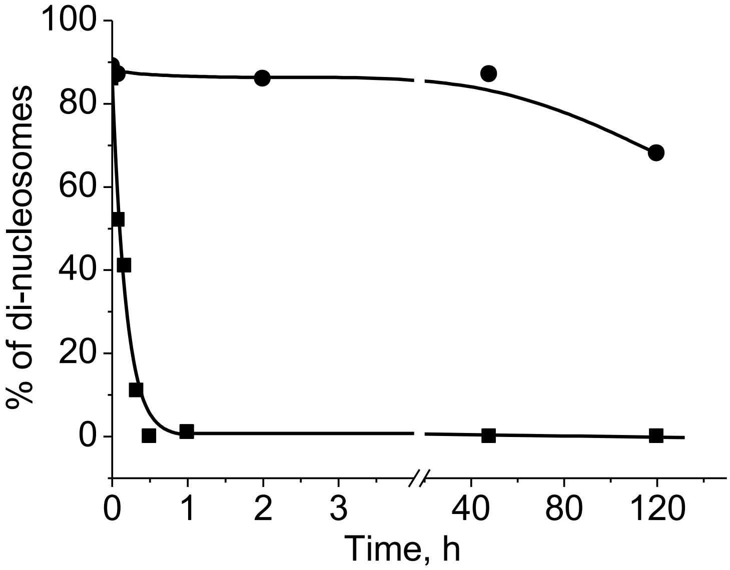

Figure S2. The yield of di-nucleosomes as a function of time, after incubation in 10 mM Tris-HCl buffer (pH 7.5) in the absence of CHAPS (squares) and in the presence of 1.6 mM CHAPS (circles). The di-nucleosome sample was diluted to 0.8 nM and incubated at 4°C for a defined time. The yield of the nucleosome sample calculated for the analysis of AFM images. The total number of di-nucleosome and DNA in the analysis for each data set was 100 molecules.

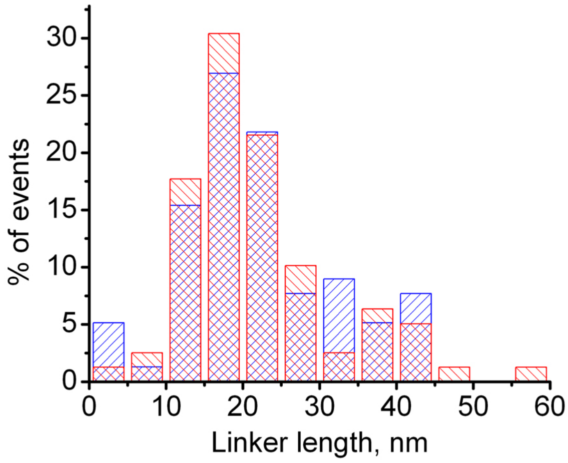

Figure S3. The distributions of the linker lengths for the control sample (no CHAPS, blue bars) and the di-nucleosome sample containing 1.6 mM CHAPS at zero incubation time.

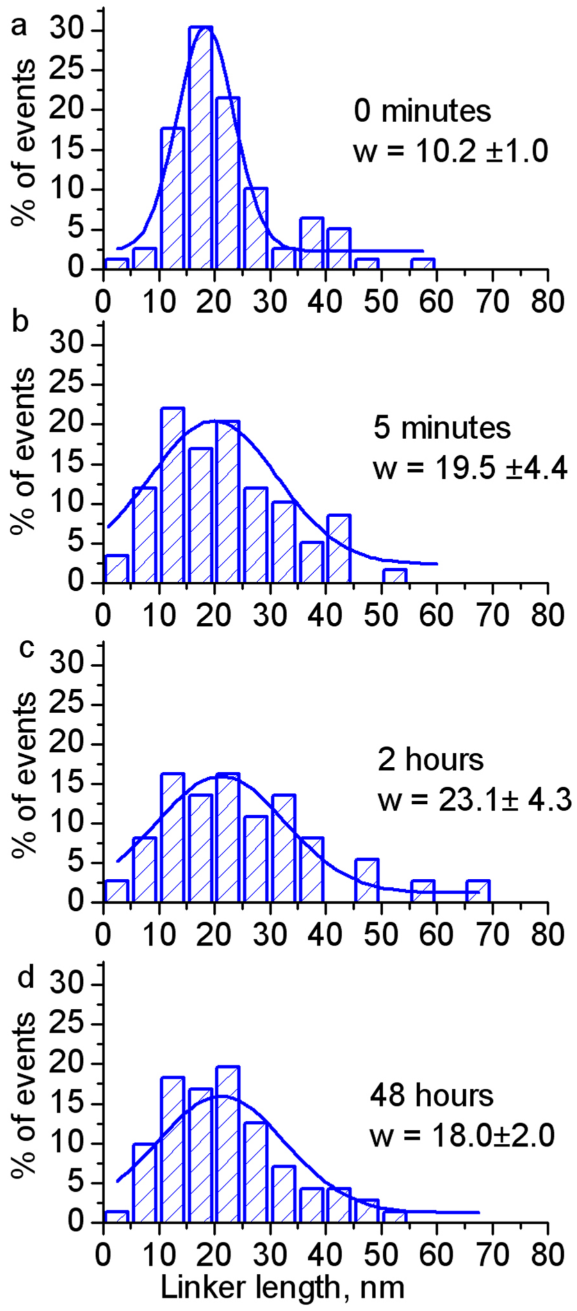

Figure S4. The linker lengths distributions for the di-nucleosome sample incubated with CHAPS during various times indicated in the figures. The widths of the histograms were 10.2 ±1.0 nm, 19.5 ±4.4 nm, 23.1± 4.3 nm and 18.0±2.0 nm for these samples incubated for 0 min, 5 min, 2 hours and 48 hours respectively.
